# Influence of Phosphorus and Cell Geometry on the Fractionation of Sulfur Isotopes by Several Species of *Desulfovibrio* during Microbial Sulfate Reduction

**DOI:** 10.3389/fmicb.2017.00890

**Published:** 2017-05-29

**Authors:** Shikma Zaarur, David T. Wang, Shuhei Ono, Tanja Bosak

**Affiliations:** Department of Earth, Atmospheric and Planetary Sciences, Massachusetts Institute of TechnologyCambridge, MA, United States

**Keywords:** sulfate reducing bacteria, organic substrate, phosphate limitation, sulfur isotopes, sulfur cycle

## Abstract

We investigated the influence of organic substrates and phosphate concentration on the rates of dissimilatory microbial sulfate reduction and the ^34^S/^32^S isotopic fractionation produced by several *Desulfovibrio* species. Our experiments corroborate the previously reported species-specific correlation between sulfur isotope fractionation and cell-specific sulfate reduction rates. We also identify cell size as a key factor that contributes to the species-effect of this correlation. Phosphate limitation results in larger cells and contributes to a small decrease in sulfur isotope fractionation concomitant with an apparent increase in cell-specific sulfate reduction rates. Sulfur isotope fractionation in phosphate-limited cultures asymptotically approaches a lower limit of approximately 5‰ as cell-specific sulfate reduction rates increase to >100 fmol cell^−1^ day^−1^. These experimental results test models that link the reversibilities of enzymatic steps in dissimilatory sulfate reduction to sulfur isotope fractionation and show that these models can provide consistent predictions across large variations in physiological states experienced by sulfate reducing bacteria.

## Introduction

The oxidation of organic matter by sulfate reducing bacteria (SRB) is a globally distributed anaerobic process that influences the redox state of the Earth's surface and the preservation of organic matter in sediments (Jørgensen, 1982; Westrich and Berner, 1984; Widdel and Hansen, 1992; Shen and Buick, 2004). Microbial sulfate reduction (MSR) fractionates sulfur isotopes, producing sulfide depleted in heavier isotopes. The magnitude of this fractionation (^34^ε, defined in Equation 6) is a critical parameter for reconstructions of the carbon and sulfur cycles through Earth history (Holland, 1973; Garrels and Lerman, 1981; Berner, 2001; Luo et al., 2016). For over half a century, many investigators have attempted to understand factors that affect sulfur isotope fractionation during MSR. Dozens of studies have reported fractionations produced in pure cultures of sulfate reducing organisms under controlled laboratory conditions (e.g., Harrison and Thode, 1958; Thode et al., 1961; Kaplan and Rittenberg, 1964; Chambers et al., 1975; Chambers and Trudinger, 1979), in experiments with natural microbial populations (e.g., Canfield, 2001; Canfield et al., 2010) and under *in situ* conditions (e.g., Jorgensen, 1979; Rudnicki et al., 2001; Wortmann et al., 2001).

Marine sulfides exhibit a wide range of sulfur isotope compositions, ranging up to a 70‰ depletion in the ^34^S/^32^S ratio relative to that of the sulfate from which they were formed (Thode et al., 1953; Kaplan et al., 1963; Canfield and Teske, 1996). Because early laboratory studies could not reproduce fractionations of this magnitude in pure culture, they established a limit of 47‰ for single-step microbially mediated sulfate reduction (Kaplan and Rittenberg, 1964). Thus, for many decades, large fractionations (>47‰) between sulfate and sulfide in rocks and natural environments were interpreted as evidence for oxidative recycling of sulfides or microbial disproportionation of intermediate sulfur species (e.g., Canfield and Thamdrup, 1994; Habicht and Canfield, 2001; Johnston et al., 2005). Recently, one bacterium (*Desulfovibrio* sp. DMSS-1), has been shown to produce fractionations that exceeded the 47‰ limit (Sim et al., 2011a). The largest fractionations (up to 66‰) are produced during slow growth with glucose as the electron donor. This approaches the theoretical limit of 70‰ proposed for microbial sulfate reduction by Brunner and Bernasconi (2005).

In pure cultures, factors reported to influence the isotopic fractionation include temperature (Canfield et al., 2006), sulfate concentration (Canfield et al., 2000; Habicht et al., 2002; Bradley et al., 2015), availability and chemical properties of the electron donor (Harrison and Thode, 1958; Kaplan and Rittenberg, 1964; Chambers et al., 1975; Kleikemper et al., 2004; Sim et al., 2011a,b; Leavitt et al., 2013; Bradley et al., 2015; Antler et al., 2017), nutrient limitation (Sim et al., 2012), sulfate reduction rate (e.g., Harrison and Thode, 1958), altered gene expression (Sim et al., 2013; Leavitt et al., 2016), and unspecified strain-specific effects (Brüchert et al., 2001; Detmers et al., 2001). These factors can be mutually dependent. For example, when the type and concentration of electron donor (hydrogen or organic carbon compounds) dictate the cell-specific sulfate reduction rate (csSRR; per-cell respiration rate) in pure culture experiments, ^34^ε appears to be inversely proportional to csSRR (Chambers et al., 1975; Kleikemper et al., 2004; Hoek et al., 2006; Sim et al., 2011b; Leavitt et al., 2013). However, changes in bacterial species (Detmers et al., 2001) or growth temperature (Kaplan and Rittenberg, 1964; Canfield et al., 2006; Johnston et al., 2007) disrupt the correlation between ^34^ε and csSRR. This led Detmers et al. (2001) to hypothesize that growth on different electron donors would result in distinct ^34^ε vs. csSRR correlations for each species.

Here, we test this hypothesis by measuring sulfur isotope fractionations produced during dissimilatory sulfate reduction by several *Desulfovibrio* species (*D. fructosovorans, D. inopinatus*, and DMSS-1) grown in batch cultures on various substrates. We also examine the effect of phosphate limitation on sulfur isotope fractionation by SRB. Phosphate is an essential nutrient and a component of DNA, RNA, lipid membranes, and the energy carrier ATP that is used to activate sulfate during MSR (Peck, 1960; Cypionka, 1995; Muyzer and Stams, 2008). Its concentrations in oceanic and sedimentary environments may limit the growth of sulfate reducing bacteria in some natural environments (Bosak et al., 2016) and may have varied dramatically through Earth's history (Planavsky et al., 2010). Therefore, limitation by this essential nutrient may directly influence both the rates of sulfate reduction and the observed sulfur isotope fractionation.

Our results confirm a strong dependence of ^34^ε on organic substrates. The data are generally consistent with an inverse correlation between ^34^ε and csSRR. Offsets and deviations among SRB species appear to be rooted in morphological and physiological differences. Further offsets from the general inverse correlation may arise from intracellular responses to phosphorus starvation (Bosak et al., 2016). Comparisons of our data with predictions from recent efforts to numerically simulate isotopic signals produced via dissimilatory sulfate reduction (Wing and Halevy, 2014) test the predictions of these models over a range of conditions that sulfate reducing bacteria can experience in nature.

## Methods

### Cultures

Three SRB species were used in this study: *Desulfovibrio inopinatus, Desulfovibrio fructosovorans*, and *Desulfovibrio* sp. DMSS-1. As is common in studies of isotope fractionation by SRB, experiments were conducted in batch cultures. Isotope fractionation trends that were first identified from batch cultures have been confirmed by chemostat experiments in which steady-state conditions can be maintained (Sim et al., 2011a,b; Antler et al., 2017). Therefore, we consider batch cultures suitable for this study. Experimental cultures were grown on different substrates (lactate, fructose, malate, and/or pyruvate) and/or in media with different initial phosphate concentrations (from <1 μM to >1 mM) and with excess sulfate as described below.

#### Desulfovibrio inopinatus

*Desulfovibrio inopinatus* (cat. no. 10711) was obtained from the German Collection of Microorganisms and Cell Cultures (DSMZ, Braunschweig, Germany: Catalogue of strains 1993). This microbe was isolated from marine sediments of Venice, Italy (Reichenbecher and Schink, 1997). Cultures were maintained anaerobically in glass serum bottles capped with butyl rubber septa under an atmosphere containing 80% N_2_ and 20% CO_2_. Culture medium was prepared according to DSMZ recipe #196-13871, with the exception of NaCl concentration because *D. inopinatus* grew better at 21 g/L than at the 7 g/L concentration suggested in the DSMZ recipe. The medium contains (per liter): NaHCO_3_, 9.0 g; Na_2_SO_4_, 3.0 g; KH_2_PO_4_, 0.20 g; NH_4_Cl, 0.30 g; NaCl, 21 g; KCl, 0.50 g, MgCl_2_·6H_2_O, 3.1 g; CaCl_2_·2H_2_O, 0.15 g; resazurin, 1 mg; a trace element solution (SL-10), 1 ml (Widdel and Pfennig, 1981; Imhoff-Stuckle and Pfennig, 1983), and a general vitamin mix (described in DSMZ recipe #141), 10 ml. Sodium ascorbate (1.5 g/L) was added to maintain anoxic conditions (Kligler and Guggenheim, 1938). The medium did not contain any yeast extract. The pH of the medium was adjusted to 7.5 by dropwise addition of 1 M NaOH or 1 M HCl prior to inoculation.

Culture medium was supplemented with lactate (28 mM), malate (28 mM), or fructose (14 mM) as organic substrate and sole electron donor. Initial concentrations of organic substrates were chosen such that sulfate would be in excess: sulfate reduction with stoichiometric conversion of any substrate to acetate would produce only ~14 mM of sulfide from the initial 21 mM sulfate, see Table 1. For each experiment, 7–15 bottles were inoculated with 5% (v/v) of a mid- to late-exponential phase pre-culture grown on the same organic substrate as that used in the experiment. Inoculums were pelleted via centrifugation and rinsed three times with clean medium in an anaerobic chamber (under an atmosphere of 80% N_2_, 15% CO_2_, and 5% H_2_) to ensure the removal of all residual sulfide and organic substrate, and then transferred into 40 mL of fresh media. Bottles were incubated in the dark at room temperature. Microbial growth was monitored daily in one “master” bottle to minimize puncturing of septa of the experimental bottles. At each time point (Tables 2, 3), one experimental bottle was subsampled for cell counts and the colorimetric assay of sulfide concentration. The remaining culture volume was sacrificed by the addition of a 1 M zinc acetate solution (at a 10% v/v ratio) to terminate microbial activity and precipitate sulfide as ZnS for isotopic analyses.

**Table 1:** **Reaction stoichiometry of and Gibbs free energy change of reaction at biochemical standard state (Δ_r_*G*°′) and at *f* = 0.90 (Δ_r_*G*′_0.90_) for dissimilatory sulfate reduction via incomplete oxidation of lactate, malate, pyruvate, or fructose to acetate**.

**Substrate**	**Reaction**	**Δ_r_*G*°′ (kJ mol^−1^)^a^**	**Δ_r_*G*′_0.90_ (kJ mol^−1^)^b^**
Lactate	2 CH_3_CH(OH)COO^−^ + ${\text{SO}}_{4}^{2-}$ ⇌ 2 CH_3_COO^−^ + 2 ${\text{HCO}}_{3}^{-}$ + HS^−^ + H^+^	−170.7	−193.5
Malate	2 CO(O^−^)CH_2_CH(OH)COO^−^ + ${\text{SO}}_{4}^{2-}$ ⇌ 2 CH_3_COO^−^ + 4 ${\text{HCO}}_{3}^{-}$ + HS^−^ + H^+^	−209.3	−242.5
Pyruvate	4 CH_3_COCOO^−^ + ${\text{SO}}_{4}^{2-}$ ⇌ 4 CH_3_COO^−^ + 4 ${\text{HCO}}_{3}^{-}$ + HS^−^ + 3 H^+^	−340.1	−379.8
Fructose	C_6_H_12_O_6_ + ${\text{SO}}_{4}^{2-}$ ⇌ 2 CH_3_COO^−^ + 2 ${\text{HCO}}_{3}^{-}$ + HS^−^ + 3 H^+^	−360.4	−391.7

*Reaction stoichiometry from Cord-Ruwisch et al. (1986), Thauer et al. (1977), and Sim et al. (2011b). Values of Δ_r_G°' are given at 298 K and 1 bar for a hypothetical ideal-dilute solution with unit activity of solutes at concentrations of 10^−7^ M for H^+^ and 1 M for all other aqueous species*.

a *Standard Gibbs energies were calculated using the CHNOSZ software package (Dick, 2008) from thermodynamic data (Shock and Helgeson, 1988; Shock, 1995; Amend and Plyasunov, 2001; Wagner and Pruß, 2002; Dalla-Betta and Schulte, 2009) compiled in database updates to the SUPCRT92 program developed by Johnson et al. (1992)*.

b *Gibbs energies of reaction were calculated at 25°C for medium compositions resembling those used for D. fructosovorans (see Methods), assuming 10% conversion of initial ${\text{SO}}_{4}^{2-}$ to HS^−^ with stoichiometric changes in concentrations of other reactants and products. Activity coefficients were assumed to be unity and pH was held constant at 7.0. All dissolved inorganic carbon was assumed to be as ${\text{PO}}_{4}^{3-}$, and all sulfide as HS^−^*.

**Table 2:** **Physiological and isotopic data for batch cultures of *D. fructosovorans* and *D. inopinatus* grown on various substrates under non-phosphate-limited conditions**.

**Substrate**	**${\text{PO}}_{4}^{3-}$ (μM)**	**Time^a^ (days)**	**OD^b^**	***N* (10^6^ cells/ml)^c^**	**ΣH_2_S (mM)**	***k* (day^−1^)**	***Y* (10^6^ cells/μmol ${\text{SO}}_{4}^{2-}$)**	**csSRR (fmol/cell/day)**	***f***	**δ^34^S sulfate**	**δ^34^S sulfide**	**^34^ε (o)**
***D. fructosovorans***												
Lactate	2,200	0.5	0.044	3.3	0.1				0.996	(−0.4)		
		3.4^*^	0.084	7.3	1.7				0.917			
		4.4	0.120	20.5	3.3	1.09 ± 0.22	8.5 ± 2.9	129 ± 34	0.843		−13.8	14.6 ± 0.8
		5.3	0.179	48.0	5.7	1.03 ± 0.12	10.2 ± 2.4	101 ± 19	0.727		−12.0	13.7 ± 0.9
		6.1	0.216	64.5	7.8	0.83 ± 0.08	9.5 ± 2.0	88 ± 15	0.629		−10.7	13.2 ± 1.0
		6.8^d^	0.212	96.0	7.6				0.636		−10.6	12.9 ± 1.0
Pyruvate	2,200	0.5	0.043	3.6	0.0				0.999	(−0.4)		
		3.4^*^	0.071	7.9	0.4				0.979		−21.0	20.9 ± 0.7
		4.4	0.141	15.3	0.9	0.69 ± 0.22	14.9 ± 6.1	46 ± 11	0.955			
		5.3	0.249	45.7	2.1	0.96 ± 0.12	23.0 ± 5.2	42 ± 7	0.901		−25.1	26.0 ± 0.8
		6.1	0.364	81.4	2.9	0.88 ± 0.08	29.7 ± 6.1	30 ± 5	0.861			
		6.9^d^	0.414	65.7	3.8				0.821		−24.9	27.1 ± 0.9
Fructose	2,200	0.5	0.043	1.4	0.0				0.998	(−0.4)		
		3.4^*^	0.071	5.5	0.4				0.983		−24.0	23.8 ± 0.7
		4.4	0.112	23.8	0.6	1.54 ± 0.22	76.3 ± 28.9	20 ± 7	0.972		−27.7	27.7 ± 0.7
		5.3	0.143	56.4	1.1	1.27 ± 0.12	72.6 ± 17.1	18 ± 4	0.950		−28.8	29.1 ± 0.7
		6.1	0.293	106.2	2.3	1.13 ± 0.08	50.5 ± 10.0	22 ± 4	0.888		−29.1	30.5 ± 0.8
		6.8^d^	0.413	60.0	4.1				0.805		−27.5	30.2 ± 0.9
***D. inopinatus***												
Fructose	1,500	0.0	0.002	0.5	0.1				0.995	(−0.9)		
(Expt. 1)		2.0^*^	0.021	0.3	0.4				0.981		−18.9	18.2 ± 0.7
		5.3	0.061		1.9				0.911			
		6.9	0.108		3.8				0.821			
		9.1	0.200		5.3				0.747			
		13.2	0.267	38.1	7.4	0.45 ± 0.02	5.4 ± 1.0	82 ± 14	0.647		−25.5	30.6 ± 1.3
		15.2	0.351	39.6	8.6	0.38 ± 0.02	4.8 ± 0.9	80 ± 13	0.590			
		19.1	0.410	46.0	8.2	0.30 ± 0.01	5.8 ± 1.1	52 ± 9	0.608		−23.1	28.6 ± 1.4
Fructose	1,500	0.0	0.002	0.1	0.1				0.997	(−0.9)		
(Expt. 2)		3.0	0.004		0.0				0.998			
		7.1	0.020		0.5				0.977			
		9.9^*^	0.017	1.4	0.8				0.964		−30.5	30.1 ± 0.7
		13.0	0.175		4.2				0.801			
		14.2	0.161		4.3				0.797			
		16.0	0.227	27.1	6.6	0.49 ± 0.03	4.4 ± 0.9	111 ± 18	0.686		−26.7	31.1 ± 1.2
		17.0	0.288		7.5				0.642			
		19.8	0.418	56.9	9.4	0.38 ± 0.02	6.4 ± 1.2	59 ± 10	0.553		−24.6	32.1 ± 1.7
Malate	1,500	0.0	0.003		0.0				1.000	(+1.0)		
		7.8	0.003	0.7	0.1				0.997			
		12.8^*^	0.009	1.0	1.6				0.924		−25.3	27.3 ± 0.8
		23.8	0.137	9.6	10.8	0.21 ± 0.02	0.9 ± 0.2	223 ± 36	0.485		−23.6	35.6 ± 2.3
		27.8	0.207	22.7	15.0	0.21 ± 0.01	1.6 ± 0.3	130 ± 21	0.284			
		32.8	0.263	(25.1)	15.8	0.16 ± 0.02	1.7 ± 0.6	96 ± 24	0.246		−17.8	40.0 ± 7.4
		35.7^d^	0.237	(22.6)	18.4				0.122			
		42.9^d^	0.278	25.0	18.4				0.124			
Lactate	1,500	0.0			0.6^e^				1.000	(−0.9)		
		1.0	0.012	0.8	0.8				0.992		−6.5	
		2.0	0.008		1.0				0.980			
		3.8	0.008		3.2				0.874			
		4.9^*^	0.012	2.2	4.3				0.823		−9.4	10.0 ± 0.9
		5.9	0.028	(2.9)	5.9	0.29 ± 0.34	0.7 ± 1.1	397 ± 296	0.746			
		7.0	0.039	(5.3)	7.3	0.42 ± 0.16	1.3 ± 0.8	329 ± 131	0.679			
		7.9	0.085	(15.4)	8.8	0.64 ± 0.11	3.4 ± 1.5	191 ± 62	0.608			
		8.9	0.131	26.0	10.6	0.61 ± 0.05	4.2 ± 1.1	146 ± 34	0.525		−10.0	13.0 ± 1.2
		11.0	0.183	28.2	10.7	0.42 ± 0.03	4.5 ± 1.2	93 ± 21	0.520		−7.6	9.6 ± 1.2
		11.9	0.199	(40.0)	9.7	0.42 ± 0.05	7.8 ± 3.0	53 ± 17	0.565			
		12.9	0.256	55.9	13.8	0.40 ± 0.03	6.0 ± 1.4	67 ± 13	0.371		−6.8	10.1 ± 1.7
		13.9^d^	0.248	(50.5)	13.1				0.406			

*Sulfur isotope values (δ^34^S) are reported with respect to V-CDT in permil (o). The δ^34^S values of initial sulfate are listed in parentheses. The initial concentration of sulfate was 21 ± 0.5 mM. Errors in k, Y, csSRR, and ^34^ε were propagated from uncertainties (1σ) associated with measurements of sulfide concentration (ΣH_2_S, ±10%), cell density (N, ±15%), and δ^34^S (±0.5‰) by standard methods (Ku, 1969)*.

a Time points marked with an asterisk (^*^) were taken to be the beginning of exponential growth (t_1_, see Calculations).

b *Optical density measured at 630 nm for D. fructosovorans and 660 nm for D. inopinatus*.

c *Cell densities shown in parentheses are extrapolated from optical density data calibrated to microscopy-based cell counts*.

d *These time points represent cultures that have reached stationary phase, and as such, k, Y, and csSRR are not listed*.

e *Sulfide (0.6 mM) was carried over from the preculture of the D. inopinatus cultures grown on lactate with 1,500 μM ${\text{PO}}_{4}^{3-}$. The presence of this initial sulfide and its isotopic composition was accounted for in the calculation of f and ^34^ε for this experiment*.

**Table 3:** **Physiological and isotopic data for batch cultures of *D. inopinatus* and DMSS-1 grown on lactate in the presence of varying concentrations of phosphate (${\text{PO}}_{4}^{3-}$)**.

**Substrate**	**${\text{PO}}_{4}^{3-}$ (μM)**	**Time^a^ (days)**	**OD^b^**	***N* (10^6^ cells/ml)^c^**	**ΣH_2_S (mM)**	***k* (day^−1^)**	***Y* (10^6^ cells/μmol ${\text{SO}}_{4}^{2-}$)**	**csSRR (fmol/cell/day)**	***f***	**δ^34^S sulfate**	**δ^34^S sulfide**	**^34^ε (o)**
***D. inopinatus***												
Lactate	150	0.0		(2.0)	0.0				1.000	(+0.9)		
		7.0^*^	0.021	5.2	3.0				0.858			
		9.8	0.079	(13.5)	12.4	0.34 ± 0.12	0.9 ± 0.5	388 ± 89	0.408			
		10.9	0.105	(17.5)	16.4	0.31 ± 0.05	0.9 ± 0.2	339 ± 56	0.219			
		12.9	0.164	(25.2)	16.6	0.26 ± 0.06	1.5 ± 0.6	180 ± 42	0.210		−5.8	16.0 ± 4.1
		16.0	0.197	(29.7)	17.0	0.19 ± 0.04	1.7 ± 0.7	111 ± 26	0.188			
		17.8^d^	0.192	(29.0)	16.8				0.201			
		20.8^d^	0.183	27.5	17.5				0.167			
		21.8^d^	0.196	(29.6)	15.4				0.267			
	15	0.0		(0.4)	0.0				1.000	(+0.9)		
		6.0	0.016	(1.4)	0.5				0.977			
		9.9^*^	0.022	0.9	2.7				0.869			
		12.8	0.039	(2.9)	5.7	0.40 ± 0.11	0.7 ± 0.3	577 ± 165	0.729			
		16.8	0.099	8.4	15.4	0.33 ± 0.03	0.6 ± 0.1	547 ± 91	0.267		−6.2	14.8 ± 2.9
		19.8	0.115	(7.9)	17.6	0.22 ± 0.03	0.5 ± 0.2	463 ± 111	0.164			
		20.8	0.138	(9.4)	16.3	0.22 ± 0.03	0.6 ± 0.2	341 ± 83	0.224			
		26.8^d^	0.114	8.5	17.1				0.187			
		28.0^d^	0.226	14.0	17.3				0.178			
	3	0.0			0.0				1.000	(+0.9)		
		6.9	0.006		0.0				0.998			
		8.8^*^	0.008	0.7	0.1				0.996			
		12.8	0.020	0.8	1.1	0.03 ± 0.05	0.1 ± 0.2	311 ± 50	0.948			
		15.7	0.031	(4.5)	3.0	0.26 ± 0.05	1.3 ± 0.5	203 ± 46	0.857			
		20.8	0.026	(3.4)	3.3	0.13 ± 0.03	0.8 ± 0.3	155 ± 34	0.841		−6.6	8.2 ± 0.8
		22.7	0.033	(4.9)	3.9	0.14 ± 0.02	1.1 ± 0.4	125 ± 28	0.814			
		25.7^d^	0.028	3.8	4.9				0.768			
		26.9^d^	0.020	(2.2)	2.2				0.896			
	<1	0.0	0.001		0.0				1.000	(+0.9)		
		8.0^*^	0.010	0.3	0.3				0.984			
		9.9^e^	0.016	(0.4)	0.7	0.14 ± 0.18	0.2 ± 0.3		0.966			
		11.9^e^	0.012	(0.3)	1.0	0.02 ± 0.09	0.0 ± 0.2		0.953			
		16.0^e^	0.016	(0.3)	1.3	0.03 ± 0.04	0.1 ± 0.1		0.940		−6.1	7.3 ± 0.8
		19.8^e^	0.014	(0.3)	1.3	0.02 ± 0.03	0.1 ± 0.1		0.940			
		20.8^e^	0.014	0.3	1.0	0.01 ± 0.02	0.1 ± 0.1		0.954			
		26.8^e^	0.022	(0.4)	2.3	0.02 ± 0.02	0.1 ± 0.1		0.893			
***Desulfovibrio*** **sp. DMSS-1**												
Lactate	360	0.0			0.0				1.000	(+1.1)		
		3.1^*^	0.006	2.9	0.8				0.964			
		4.2	0.067	(12.2)	6.6	1.31 ± 0.28	1.6 ± 0.7	824 ± 180	0.684			
		5.0	0.072	(12.9)	9.7	0.76 ± 0.16	1.1 ± 0.5	676 ± 147	0.538		−4.8	8.2 ± 1.1
		6.1	0.111	18.8	10.7	0.61 ± 0.05	1.6 ± 0.3	379 ± 55	0.493			
	36	0.0			0.0				1.000	(+1.1)		
		3.1^*^	−0.004	1.3	0.8				0.962			
		4.2	0.060	(11.1)	5.2	1.91 ± 0.30	2.2 ± 0.8	862 ± 204	0.753		−3.0	4.8 ± 0.9
		5.0	0.095	(16.4)	12.0	1.26 ± 0.17	1.3 ± 0.5	939 ± 223	0.430			
		6.1^d^	0.095	(16.4)	11.3				0.461			
		7.0^d^	0.095	(16.4)	10.9				0.480			
	5	0.0	0.001	(2.2)	0.0				1.000	(+1.1)		
		4.0^*^	0.006	(3.0)	1.0				0.950			
		6.0	0.016	(4.5)	2.2	0.21 ± 0.20	1.3 ± 1.3	165 ± 47	0.894		−3.9	5.3 ± 0.8
		13.0	0.038	(7.7)	7.7	0.11 ± 0.04	0.7 ± 0.4	149 ± 35	0.631			
		14.9^d^	0.036	(7.5)	8.2				0.612			
		20.8^d^	0.037	(7.6)	11.7				0.445			
	<1	0.0		(1.8)	0.0				1.000	(+1.1)		
		7.9	0.001	(2.2)	0.0				1.000			
		13.0	0.000	(1.8)								
		14.8^*^	0.000	(2.1)	0.0				0.999			
		21.7^e^	0.000	(1.6)	0.0	−0.04 ± 0.05	227 ± 7216		1.000			
		28.9^e^	0.000	(2.0)	0.0	0.00 ± 0.03	−1.6 ± 26.9		0.998			
		35.7^e^	0.002	(2.4)	0.3	0.01 ± 0.02	1.0 ± 3.3		0.986			
		42.8^e^	0.001	(2.2)	0.2	0.00 ± 0.01	0.6 ± 4.4		0.991		−4.7	5.9 ± 0.7

*See caption of Table 2 for explanations of data columns*.

a Time points marked with an asterisk (^*^) were taken to be the beginning of exponential growth (t_1_, see Calculations).

b *Optical density measured at 660 nm*.

c *Cell densities shown in parentheses are extrapolated from optical density data calibrated to microscopy-based cell counts*.

d *These time points represent cultures that have reached stationary phase, and as such, k, Y, and csSRR are not listed*.

e *No growth and/or sulfide production was detected. Uncertainties in k and/or Y exceed the values themselves; therefore, csSRRs calculated for these time points would be spurious and are not listed*.

To test the effect of phosphate limitation, additional batch cultures were grown with varying initial concentrations of phosphate in the medium. Serum bottles used for these cultures were autoclaved three times with deionized water (18.2 MΩ·cm, Barnstead Nanopure™ filtration system) to remove residual phosphate. Medium was prepared as described above, with lactate (28 mM) as organic substrate and without added phosphate. Phosphate (as KH_2_PO_4_) was then added from a concentrated stock solution, to final concentrations of 150, 15, and 3 μM. Another set of serum bottles did not receive any added phosphate. The concentration of phosphate in this nominally “0” phosphate medium was estimated to be <1 μM based on lot analyses provided with the chemicals used in the media (see also Experiments with Different Phosphate Concentrations). Figure A2 shows that ~3 to 5 μM of added phosphate is limiting, suggesting that the blank is likely <5 μM. Conclusions reached in this paper would be unaffected by a blank of 5 μM or less. Experimental bottles (containing 10 mL of fresh media) were inoculated with a 5% (v/v) sample of pre-cultures that were already conditioned to growth in the presence of lower or limiting concentrations of phosphate. Cell densities were low when phosphate was limiting, so inoculums were pelleted and rinsed only once to minimize the loss of biomass prior to inoculation. Cultures were monitored and sampled as described above.

#### Desulfovibrio fructosovorans

*Desulfovibrio fructosovorans* strain JJ (cat. no. 3604) was obtained from DSMZ. This strain was isolated from estuarine sediment (Jones et al., 1984; Cord-Ruwisch et al., 1986), and it incompletely oxidizes pyruvate, lactate, or fructose to acetate (Ollivier et al., 1988). The mineral medium (modified from DSMZ recipe #63) contained (per liter): NaHCO_3_, 2.6 g; Na_2_SO_4_, 3.0 g; KH_2_PO_4_, 0.30 g; NH_4_Cl, 0.50 g; KCl, 0.20 g; CaCl_2_, 0.10 g; MgCl_2_, 2.0 g; resazurin, 1 mg; SL-10 trace element solution (see above), 1 ml; vitamin solution #141 (see above), 10 ml; and sodium ascorbate, 1.5 g (see above). Media were supplemented with limiting concentrations of one of the following organic substrates: lactate, 20 mM; pyruvate, 40 mM; or fructose, 10 mM. The starting concentrations of lactate, fructose, and pyruvate were chosen such that 10 mM of sulfide would be produced by stoichiometric conversion of the substrate to acetate (Table 1). The pH of the medium was adjusted to 7.0 before inoculation. Bottles for all experiments with *D. fructosovorans* were inoculated with 5% (v/v) of a late-exponential phase pre-culture grown on lactate. Media and cultures were otherwise prepared and maintained as described for *Desulfovibrio inopinatus* above.

#### *Desulfovibrio* sp. strain DMSS-1

*Desulfovibrio* sp. DMSS-1 (henceforth “DMSS-1”), isolated from a salt marsh on Cape Cod, Massachusetts, USA and characterized by Sim et al. (2011b), was grown in batch cultures in the presence of varying concentrations of phosphate. Serum bottles were cleaned and prepared in the same manner as described above for *D. inopinatus*. Medium for DMSS-1 was prepared following Sim et al. (2011b), with the exception of phosphate, and contained (per liter): NaHCO_3_, 9 g; Na_2_SO_4_, 3 g; NH_4_Cl, 0.3 g; NaCl, 21 g; KCl, 0.5 g; MgCl_2_·6H_2_O, 6 g; CaCl_2_·2H_2_O 0.3 g; resazurin, 1 mg; SL-10 trace element solution (see above), 1 ml; vitamin solution #141 (see above), 10 ml; 1 ml of selenium stock solution (0.4 mg of Na_2_SeO_3_ per 200 ml of 0.01 N NaOH); and sodium ascorbate, 1.5 g (see above). Phosphate was added to final concentrations of 360, 36, 5 μM, and “0” (<1 μM).

### Analyses

#### Cell counts and sulfide assays

Growth was monitored using optical density (OD) measurements and microscopic cell counts. OD was measured at 630 or 660 nm using a spectrophotometer (Synergy 2 microplate reader, BioTek, Winooski, Vermont, USA). For cell counts, subsamples of experimental cultures were preserved in 2.5% glutaraldehyde at 4°C. Preserved cells were stained with SYBR Green I nucleic acid stain (Invitrogen Molecular Probes, Eugene, Oregon, USA), and filtered onto Whatman 0.2 μm Nuclepore polycarbonate filters. Stained cells were visualized and imaged by epifluorescence microscopy using a Zeiss Axio Imager M1 microscope (Carl Zeiss Microscopy, LLC), and cell densities were determined by manual counting (Noble and Fuhrman, 1998). Cell lengths and widths were measured in the epifluorescence micrographs using measuring tools in Zeiss AxioVision software.

Sulfide from subsamples was precipitated as ZnS in a 50 mM zinc acetate solution, and stored at 4°C until analysis. Sulfide concentrations (ΣH_2_S = H_2_S + HS^−^ + S^2−^) were determined by a modified methylene blue colorimetric method (Cline, 1969). Briefly, 200 μL samples of medium were reacted with 1 ml of 0.05 M zinc acetate and 10 μL of *N*,*N*-dimethyl-*p*-phenylenediamine sulfate solution. Optical density was read at 670 nm using a microplate reader (Sim et al., 2011b).

#### Sulfur isotope ratios

Sulfide was extracted for isotopic analysis by acidifying each ZnS sample with 3 to 6 M HCl, and gently boiling for 1 h under a stream of N_2_ gas. Sulfate in samples were then converted to H_2_S using a general reducing agent (HCl, HI, and H_3_PO_2_) (Thode et al., 1961; Forrest and Newman, 1977; Arnold et al., 2014). Volatiles were passed through a condenser and a distilled H_2_O trap. H_2_S produced in the reactions was precipitated as ZnS in zinc acetate, and then converted to Ag_2_S via addition of silver nitrate, or precipitated as Ag_2_S directly in a silver nitrate solution. The recovered Ag_2_S was washed with deionized water, dried at 70°C, and converted to SF_6_ by reaction with F_2_ at 300°C overnight. The SF_6_ product was purified by cryogenic trapping and preparative gas chromatography, and analyzed on a ThermoFinnigan MAT 253 isotope-ratio mass spectrometer operated in dual-inlet mode as described previously (Ono et al., 2006).

Sulfur isotope values are reported in the standard δ notation against Vienna Cañon Diablo Troilite (VCDT):

(1) δ34Ssulfide=(S34/S32)sample(S34/S32)VCDT−1

Following IUPAC recommendations (Coplen, 2011), we have omitted the factor of 1000‰ from the definition of δ in Equation 1.

### Calculations

Specific growth rates (*k*) for the cultures were calculated using an exponential growth equation (Monod, 1949):

(2) $$k_{x}=\;\frac{\ln\left(N_{x}/N_{1}\right)}{t_{x}-t_{1}}$$

where *N*_*x*_ and *N*_1_ are the cell densities (number of cells per milliliter) at *t*_*x*_ and *t*_1_, respectively (Sim et al., 2011b). Growth rates calculated in this manner represent an average of the cumulative growth of the organism over the time interval between the beginning of exponential growth (*t*_1_) and the time at which the sample was taken (*t*_*x*_).

Cellular growth yields (*Y*) were calculated with respect to amount of produced sulfide:^1^

(3) $$Y_{x}=\frac{N_{x}-N_{1}}{{\left[{\text{H}}_{2}\text{S}\right]}_{x}-{\left[{\text{H}}_{2}\text{S}\right]}_{1}}$$

The cell-specific sulfate reduction rate (csSRR) is defined as the amount of sulfate reduced per cell per unit time, and can be described as:

(4) $${\text{csSRR}}_{x}=k_{x}/Y_{x}$$

In a batch culture, isotopic fractionation factors can be determined from a Rayleigh distillation equation for closed systems. Here, we use the measured isotopic composition of the produced sulfide (δ^34^S_sulfide_) to calculate the fractionation factor α, assuming isotopic mass balance between sulfide and remaining sulfate:

(5) $$\alpha ={\left(\text{ln }f\right)}^{-1}\times \text{ln}\left[1-\left(1-f\right)\times \frac{\delta^{34}{\text{S}}_{\text{sulfide}}+1}{\delta^{34}{\text{S}}_{\text{initial}}+1}\right]$$

where δ^34^S_initial_ and δ^34^S_sulfide_ are the measured isotopic compositions of the initial sulfate in the media and the produced sulfide respectively, and *f* is the fraction of initial sulfate that remained at the time of sampling. The value of *f* is calculated from measured ΣH_2_S concentrations and assuming that all consumed sulfate was reduced into sulfide.

The isotopic enrichment factor (^34^ε) is defined as:

(6) ε34=1− α34

According to this definition, positive ^34^ε values represent depletion of ^34^S in sulfide with respect to sulfate.

## Results

### Experiments with different SRB species and substrates

**Figure A1:**
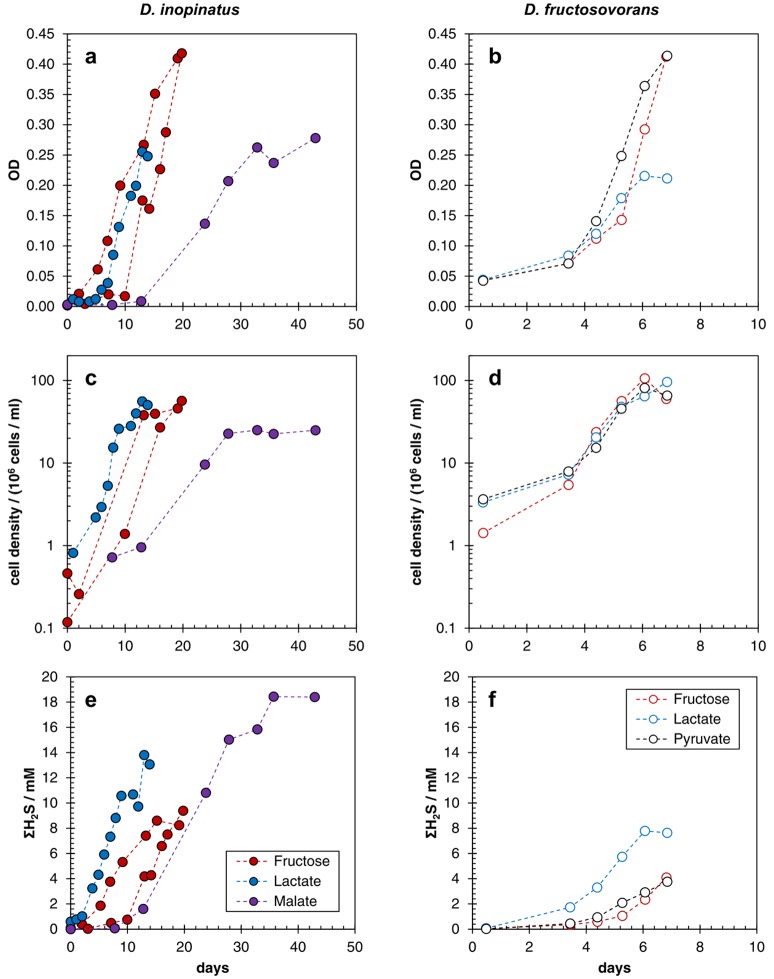
**Growth curves showing optical density of cultures (a,b)**, cell densities determined by epifluorescence microscopy **(c,d)**, and total sulfide (ΣH_2_S) concentration **(e,f)** in batch cultures of *D. inopinatus* (left) and *D. fructosovorans* (right) grown on different organic substrates (see the legend for the explanation of color coding) in phosphate-replete (>1000 μM) media. Data are from Table 2. Note the difference in scales for the time axis between left and right panels. The uncertainties in cell density and sulfide concentration, respectively, were ±15% and ±10%, respectively.

Table 2 shows data from experiments that were designed to test the dependence of sulfur isotope fractionation on species (*D. fructosovorans* and *D. inopinatus*) and substrates (lactate, fructose, malate, or pyruvate). Batch cultures used in these experiments were typically grown to late exponential phase. Cultures of *D. fructosovorans* grew in media with fructose, pyruvate, or lactate as the organic substrate. Exponential growth was observed within 2 to 3 days, and experiments lasted for 7 days (Figure A1). Growth rates (*k*) on all three substrates ranged between 0.6 and 1.5 day^−1^. Cultures of *D. inopinatus* grew in media that contained fructose, malate or lactate as the electron donors. Growth experiments with *D. inopinatus* lasted 14, 20, and 43 days, respectively, for cultures grown on lactate, fructose and malate, respectively, due to the longer lag phase (Figure A1). Growth rates of *D. inopinatus* in cultures grown on fructose and lactate were similar (0.40 and 0.44 day^−1^, respectively), but were lower during growth on malate (0.17 day^−1^). Sulfide concentrations in all cultures increased with the increasing cell densities (Figure A1). Cultures of *D. inopinatus* grown on malate produced more sulfide (up to 18 mM) than the 14 mM concentration predicted by incomplete malate oxidation (Table 1), perhaps indicating that some of the malate may have been oxidized completely to CO_2_ or to small organic compounds.

**Figure 1:**
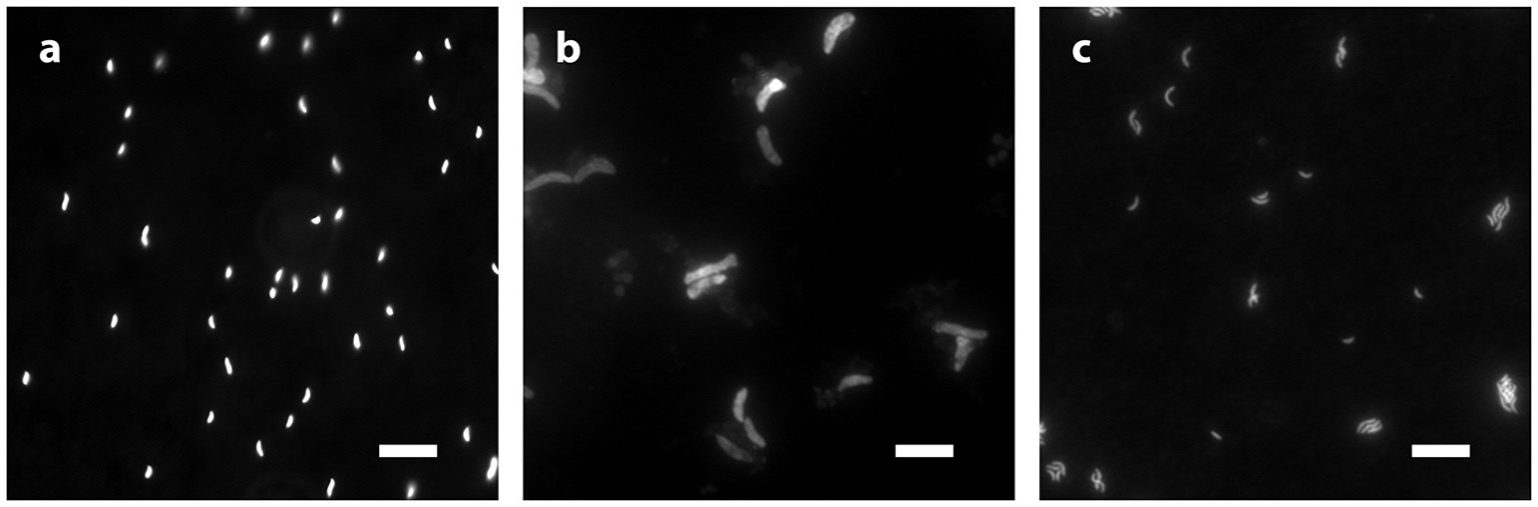
**Morphology of species growing in phosphate-rich media and visualized by epifluorescence microscopy: (a)** *D. fructosovorans* (pyruvate, 2,200 μM ${\text{PO}}_{4}^{3-}$, 6.1 days); **(b)** *D. inopinatus* (malate, 1,500 μM ${\text{PO}}_{4}^{3-}$, 27.8 days); and **(c)** DMSS-1 (lactate, 360 μM ${\text{PO}}_{4}^{3-}$, 6.1 days). Scale bars in all panels represent 10 μm.

*D. inopinatus* cells were both longer and wider than cells of *D. fructosovorans* and DMSS-1 (Figure 1). Cell morphology appeared to be mostly independent of organic substrate (Table 4), but small, measurable differences were observed in some cultures of *D. inopinatus*. Cells of *D. inopinatus* were on average ~1 μm longer when grown on malate compared to fructose or lactate, and slightly wider (by ~0.3 μm) when grown on fructose compared to malate or lactate. Cells of *D. fructosovorans* grown on different substrates did not exhibit measurable differences.

**Table 4:** **Cell sizes (values are mean ± 1σ measured on *n* > 30 cells)**.

**Strain**	**Substrate**	**${\text{PO}}_{4}^{3-}$ (μM)**	**Time (days)**	**Length (μm)**	**Width (μm)**	**Surface area (μm^2^)**	**Volume (μm^3^)**
*D. fructosovorans*	Lactate	2,200	6.1	2.7 ± 0.5	1.0 ± 0.2	10 ± 3	2.1 ± 1.0
	Pyruvate	2,200	6.1	2.5 ± 0.5	0.9 ± 0.1	8 ± 2	1.6 ± 0.7
	Fructose	2,200	6.1	2.7 ± 0.4	1.0 ± 0.1	10 ± 2	2.0 ± 0.7
*D. inopinatus*	Fructose	1,500	13.2	6.3 ± 1.1	2.1 ± 0.3	49 ± 11	22.3 ± 7.7
	(Expt. 1)		19.1	6.7 ± 1.4	2.1 ± 0.2	50 ± 12	22.7 ± 7.7
	Fructose	1,500	16.0	7.1 ± 1.4	2.3 ± 0.3	59 ± 14	29.4 ± 10.3
	(Expt. 2)		19.8	6.4 ± 1.1	1.8 ± 0.3	42 ± 11	17.2 ± 7.4
	Malate	1,500	12.8	8.1 ± 1.8	1.7 ± 0.3	48 ± 13	18.7 ± 7.6
			27.8	7.6 ± 1.3	1.7 ± 0.3	47 ± 13	18.8 ± 7.4
	Lactate	1,500	8.9	6.8 ± 1.6	1.7 ± 0.3	41 ± 8	16.2 ± 5.1
			12.9	7.0 ± 1.3	1.8 ± 0.3	45 ± 8	18.0 ± 5.5
		150	7.0	11.4 ± 4.5	1.9 ± 2.9	73 ± 102	32.1 ± 76.1
			10.9	10.0 ± 2.0	1.1 ± 0.2	38 ± 9	10.4 ± 3.5
		15	16.8	9.3 ± 2.6	1.9 ± 0.5	63 ± 23	28.7 ± 15.3
			28.0	12.4 ± 8.0	1.9 ± 0.4	79 ± 52	36.3 ± 31.5
		3	8.8	23.2 ± 13.9	1.9 ± 0.3	148 ± 96	71.1 ± 54.7
			25.7	18.8 ± 16.5	1.9 ± 0.4	125 ± 119	61.9 ± 69.6
		<1	8.0	18.8 ± 9.0	1.7 ± 0.3	106 ± 52	44.9 ± 25.7
			20.8	30.3 ± 22.3	2.0 ± 0.4	201 ± 152	102.6 ± 92.0
*Desulfovibrio* sp. DMSS-1	Lactate	3,600^a^	3	2.3 ± 0.5	−	−	−
			−	1.8 ± 0.2	−	−	−
			−	1.9 ± 0.3	−	−	−
			−	2.3 ± 0.4	−	−	−
		360	3.1	2.6 ± 1.0	0.7 ± 0.1	6 ± 2	0.9 ± 0.5
		36	5.0	3.1 ± 0.9	0.5 ± 0.1	6 ± 2	0.7 ± 0.3
		5	13.0	4.7 ± 1.4	0.7 ± 0.1	11 ± 4	1.9 ± 1.2
		<1	21.7	4.6 ± 1.5	0.7 ± 0.1	12 ± 4	2.1 ± 1.1
			42.8	6.8 ± 2.3	0.7 ± 0.1	17 ± 7	3.1 ± 1.6
*D. vulgaris* Hildenborough^b^	Pyruvate	−	−	2.9 ± 0.8	0.8 ± 0.1	9 ± 3	1.7 ± 0.7

*Surface areas and volumes were calculated for individual cells by approximating each cell as a cylinder*.

a *Data from Sim et al. (2011b) for batch and continuous cultures of DMSS-1*.

b *Data from microscopy images taken by Sim et al. (2013) of a continuous culture of D. vulgaris Hildenborough*.

Calculated sulfur isotope enrichment factors (^34^ε) ranged from 13 to 31‰ in *D. fructosovorans* cultures and from 10 to 40‰ in *D. inopinatus* cultures (Table 2). The smallest ^34^ε values (averaging 13 and 11‰ for the two species, respectively) were observed in cultures grown on lactate. When grown on fructose, both bacteria exhibited 15–17‰ larger ^34^S/^32^S fractionations. The highest enrichment factors, up to 40‰, were observed in the cultures of *D. inopinatus* grown on malate (Table 2).

### Experiments with different phosphate concentrations

**Figure A2:**
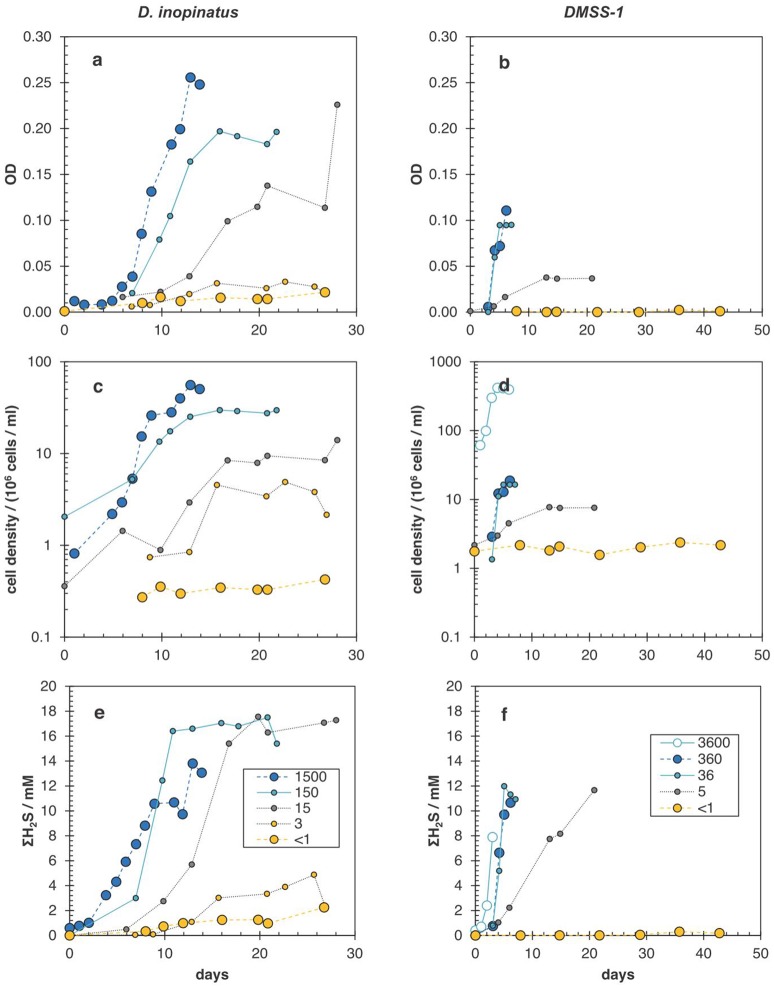
**Growth curves showing optical density of cultures (a,b)**, cell densities determined by epifluorescence microscopy **(c,d)**, and total sulfide (ΣH_2_S) concentration **(e,f)** in batch cultures of *D. inopinatus* (left) and DMSS-1 (right) grown on lactate in the presence of different initial phosphate concentrations (in μM; legend shown in bottom panels). Data are from Tables 2, 3, with the exception of the data for DMSS-1 cultures grown at 3600 μM phosphate (open circles in right panels), which are from Sim et al. (2011b). Note the difference in scales for the time axis between left and right panels. The uncertainties in cell density and sulfide concentration, respectively, were ±15% and ±10%, respectively.

Data shown in Table 3 are from experiments that test the effect of variable phosphate concentrations on *D. inopinatus* and DMSS-1. Cultures grown in media containing initial phosphate concentrations ≤15 and ≤5 μM for *D. inopinatus* and DMSS-1, respectively, had smaller growth rates and cell densities relative to cultures grown at higher phosphate levels (≥150 and ≥36 μM; Figure A2). At the lowest phosphate concentration (nominally <1 μM), the optical density of *D. inopinatus* increased slowly, but that of DMSS-1 did not increase over a period of 43 days. Sulfide was produced under all tested conditions, though *D. inopinatus* and DMSS-1, produced only up to 2.3 and 0.3 mM of sulfide, respectively, when no phosphate was added to the growth media (**Figure 4**). Growth rates and csSRR of *D. inopinatus* and DMSS-1 were the highest at 15 and 36 μM phosphate, respectively.

**Figure 2:**
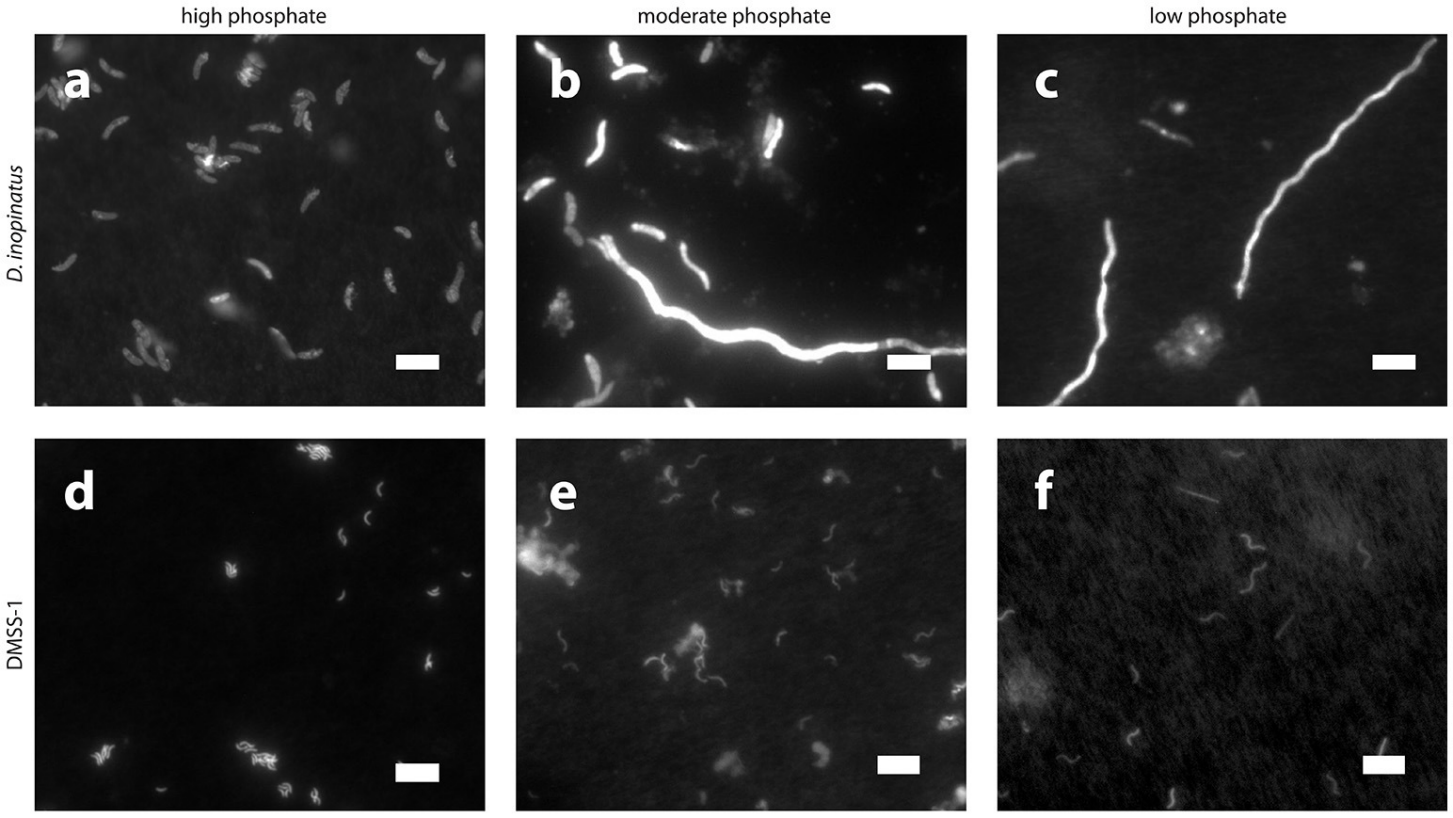
**Epifluorescence photomicrographs of cultures grown on lactate in the presence of different concentrations of phosphate**. Top row, *D. inopinatus* in **(a)** 1,500 μM ${\text{PO}}_{4}^{3-}$ (12.9 days); **(b)** 15 μM (14.7 days); and **(c)** <1 μM (8.0 days). Bottom row, DMSS-1 in **(d)** 360 μM ${\text{PO}}_{4}^{3-}$ (6.1 days, see also Figure 1c for inset); **(e)** 5 μM (13.0 days); and **(f)** <1 μM (42.8 days). Scale bars represent 10 μm. Panels **(b,c,f)** show long chain-like single cells.

**Figure 3:**
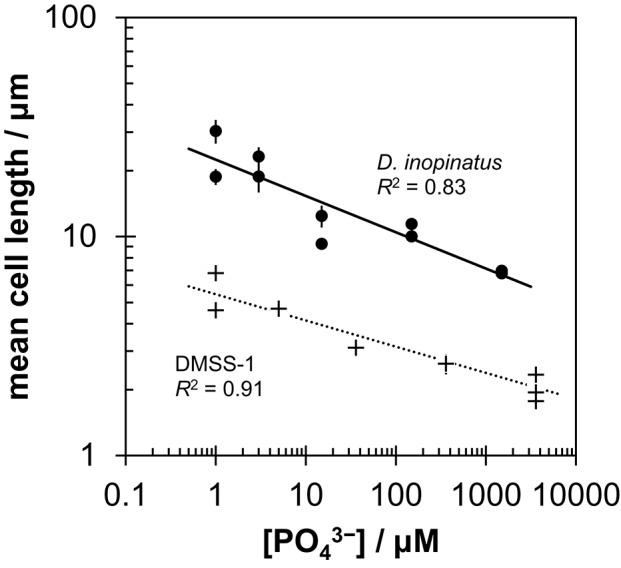
**Relationship between mean cell length and initial phosphate concentration in cultures of *D. inopinatus* (•) and DMSS-1 (+) grown on lactate**. Data are from Table 4. Vertical error bars are 1 s.e.m. (standard error of the mean). For DMSS-1, error bars are smaller than the symbols.

Low phosphate concentrations strongly influenced the cell morphology (Table 4). At the lowest phosphate condition (<1 μM), the average *D. inopinatus* cell was up to five times longer (Figure 2), whereas the average cell length of DMSS-1 increased up to three times relative to the phosphate-replete conditions. In addition, the variance (σ^2^) in cell length (Table 4) was larger at lower phosphate concentrations: cell sizes at high concentrations of phosphate were rather uniform, but both “normal” single cells with typical lengths (~7 μM) and extremely long chains (over 100 μm in *D. inopinatus* cultures, Figure 2) were present at low phosphate concentrations. The cell lengths were strongly correlated with the phosphate concentration in the cultures of both organisms (Figure 3), and cell lengths typically increased with time in each phosphate-limited experiment. Samples were vigorously vortexed prior to staining, filtration, and visualization. Because these procedures did not separate these long chain-looking cells and no cell wall was recognized, we counted them as single cells. However, microscopy-based cell counts were challenging because a single long chain-like cell (as long as 10 individual cells) can appear similar to a chain of several cells of shorter length located end-to-end (e.g., Figure 2c). These issues may cause some of the scatter in the growth rate and csSRR data, but should not affect the overall trends observed in this study.

**Figure 4:**
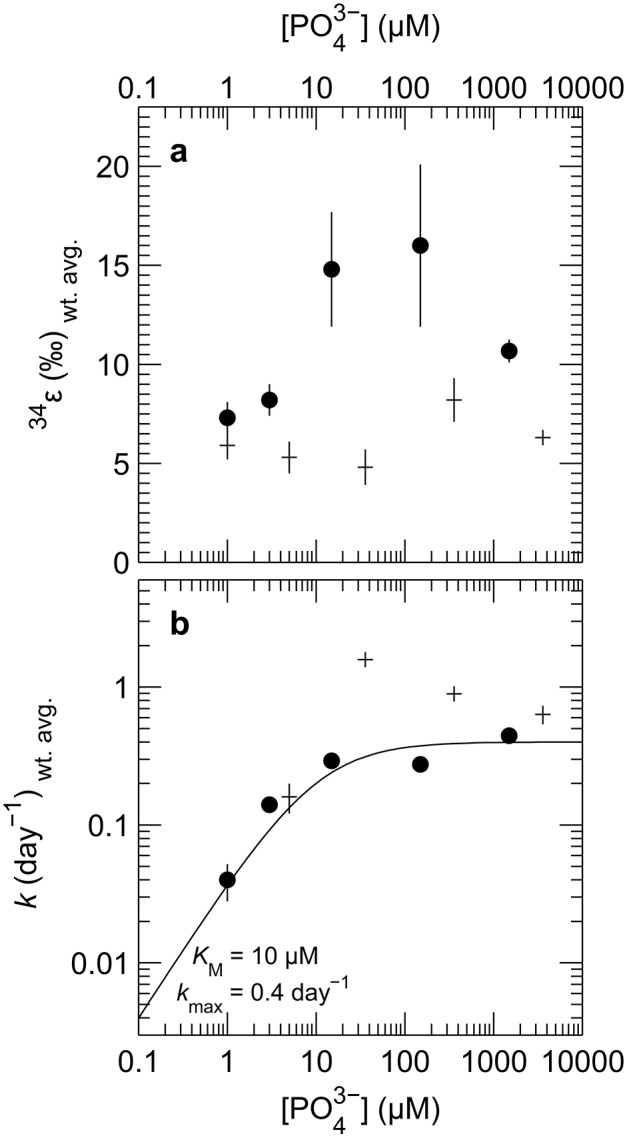
**Plot of weighted mean (a)** isotope fractionation (^34^ε) and **(b)** growth rate (*k*) for batch cultures of *D. inopinatus* (•) and DMSS-1 (+) grown on lactate at different initial concentrations of phosphate. Data from individual bottles were weighted by 1/σ^2^. Vertical error bars are 1σ for the weighted means, calculated following Bevington and Robinson (2002). Points corresponding to experiments in which phosphate was <1 μM are plotted at 1 μM. Data for DMSS-1 grown in 3,600 μM phosphate are from Sim et al. (2011b). The curve in **(b)** is a fit of the Monod equation (see Equation 7) (Monod, 1949), to the *D. inopinatus* data.

The isotopic composition of sulfide was measured during mid-exponential growth phase in most of our experiments, with the exception of the culture of DMSS-1 grown with <1 μM initial phosphate, where δ^34^S_sulfide_ was measured at 42.8 days, and growth was not detected at any point during the experiment. While measured δ^34^S_sulfide_ values varied little, from −5.8 to −6.6‰ for *D. inopinatus* and from −3 to −4.7‰ for DMSS-1, the calculated enrichment factors (^34^ε) decreased with decreasing phosphate concentrations (Figure 4a). The ^34^ε values in *D. inopinatus* cultures grown with lactate decreased from 16‰ at 150 μM phosphate (similar to regular, phosphate-replete conditions) to 7‰ at <1 μM phosphate. The ^34^ε values of DMSS-1 cultures decreased from 8 to 5‰ over a similar range of phosphate concentrations.

## Discussion

### Variation of ^34^ε with substrates and species

#### Differences among species

Both *D. fructosovorans* and *D. inopinatus* discriminate against heavier isotopes of sulfur more when they grow on fructose (^34^ε ~ 30‰) relative to the growth on lactate (10 to 15‰). Data from Sim et al. (2011b) for DMSS-1 growing on fructose (30 to 40‰) and lactate (~6‰) in batch cultures exhibited the same trend and similar ranges of ^34^ε. Growth on pyruvate and malate yielded ^34^ε values that were dissimilar among the studied species. While fractionations during the growth on pyruvate were larger than those during the growth on lactate and smaller than those during the growth on fructose for both *D. fructosovorans* and DMSS-1, ^34^ε was ~27‰ for the former species, but only 8‰ for the latter. The relatively high fractionation by pyruvate-grown *D. fructosovorans* contrasts with previous studies of sulfate reducing bacteria that oxidize organic substrates completely to CO_2_. These microbes generally produce values of ^34^ε smaller than 19‰ when oxidizing pyruvate to acetate in batch culture (Brüchert, 2004; Sim et al., 2011b). An increased fractionation may be a consequence of simultaneous fermentation and respiration of pyruvate, an effect that has been inferred in cultures of *D. vulgaris* Hildenborough and other species (Sass et al., 2002; Sim et al., 2013), and to which higher fractionations could be attributed (Sim et al., 2013). Another unexplained difference in fractionation is evident during the growth on malate by *D. inopinatus* (up to ~40‰) and DMSS-1 (ca. 17‰). Such differences suggest that the magnitude of sulfur isotope fractionations is not a direct function of the organic substrate itself, but depends on the specific pathways by which organisms take up and oxidize organic compounds, transfer the reducing equivalents to sulfate (or instead to an organic compound via fermentation), and generate ATP.

#### ^34^ε and csSRR

**Figure 5:**
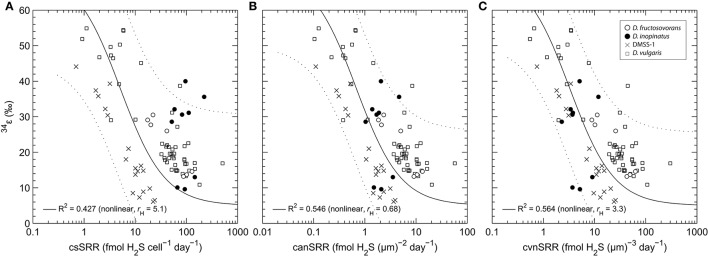
**Comparison of correlations between (a)** ^34^ε and cell-specific sulfate reduction rate (csSRR); **(b)** ^34^ε and cell area-normalized sulfate reduction rate (canSRR); and **(c)** ^34^ε and cell volume-normalized sulfate reduction rate (cvnSRR) for several *Desulfovibrio* spp. grown in culture under nutrient-replete conditions. In **(a)**, the DMSS-1 and *D. vulgaris* (Hildenborough) data are from Sim et al. (2011b) and Leavitt et al. (2013), respectively. In **(b,c)**, the sulfate reduction rates have been normalized using the cell size data shown in Table 4. The solid curve and coefficient of determination (*R*^2^) represent non-linear least squares fits of a model function through all data in each panel. Equations describing the curves are of the form: *y* = ^34^ε_max_ − *x* · (^34^ε_max_−^34^ε_min_)/(r_H_+*x*), where ^34^ε_max_ = 71‰ (equilibrium at ~20°C and pH ~7.0; (Millero et al., 1988; Otake et al., 2008)), ^34^ε_min_ = 5‰ (high-rate asymptote, see Appendix), and *r*_*H*_ is a fitted parameter that has the same units as the *x*-axis (it represents the sulfate reduction rate at which ^34^ε is halfway between its maximum and minimum limits). This equation was chosen because it can closely approximate model results of Wing and Halevy (2014) for the typical range of csSRR observed in experiments (see Appendix), and because *R*^2^ for fits to this equation are uniformly higher than for linear least squares regressions of ^34^ε against the log-transformed rates. Dotted envelope marks the non-simultaneous 95% prediction band.

The trend in ^34^ε vs. csSRR for *D. fructosovorans* generally resembles the inverse correlations reported by several previous studies (Harrison and Thode, 1958; Kaplan and Rittenberg, 1964; Chambers et al., 1975; Kleikemper et al., 2004; Hoek et al., 2006; Sim et al., 2011a,b; Leavitt et al., 2013). Data from several of these studies are shown in Figure 5a. *D. inopinatus* data fall within the range observed for other *Desulfovibrio* species, but there is no clear trend between ^34^ε and csSRR; this may be in part due to the high fractionations observed during growth on malate. In general, trends for different species are subparallel in ^34^ε–log_10_(csSRR) space, have different slopes and intercepts, and may be offset by several orders of magnitude in csSRR. These observations are evidence of the complexity that depends on species-specific responses to substrates and growth conditions.

#### Cell size and ^34^ε

The three *Desulfovibrio* species have very different cell sizes despite their grossly similar vibrioid morphology (Figure 1), and generally, higher sulfate reduction rates are observed in the larger cells. To test whether the surface areas or volumes of cells account for some of the observed differences in csSRR between species, we expressed ^34^ε–log_10_(sulfate reduction rate) relationships per unit of cell surface area or volume. To estimate the surface areas and volumes, we measured the cell dimensions of *D. inopinatus, D. fructosovorans* and DMSS-1 grown in this study and those of DvH previously grown in our laboratory (Sim et al., 2013; Table 4). The csSRR values were divided by either surface area or volume, and expressed as “cell area (or volume) normalized sulfate reduction rate” [canSRR or cvnSRR; units of fmol H_2_S (μm)^−2^ day^−1^ or fmol H_2_S (μm)^−3^ day^−1^, respectively]. Figures 5b,c show the ^34^ε data for these species plotted against sulfate reduction rates normalized in this manner. Non-linear least-squares regression of a modified Michaelis-Menten-type equation (see Figure 5 legend) to plots of ^34^ε against canSRR or cvnSRR has higher coefficients of determination (*R*^2^) of 0.55 or 0.56 (respectively), compared to 0.43 for ^34^ε against csSRR. Thus, normalizing sulfate reduction rates to either cell surface area or cell volume reduces the scatter of the data (Figure 5). The improved fits may arise from the distribution of membrane transport proteins across the cell surface, and from the constraints on the amounts of enzyme in a single cell by the cell volume (Pomeroy et al., 2007). This result underscores similarities in the isotopic signals produced per unit of membrane area or biomass of different cultured sulfate reducing microbes, in spite of their differing cell geometries, growth rates, and capacities to oxidize different electron donors. We hypothesize that the larger cells require more energy (electrons) per cell to synthesize cellular components, and therefore exhibit higher csSRR values. Normalizing the sulfate reduction rate to cell volume eliminates that inherent difference amongst microbial species. Alternatively or additionally, larger cells also have larger surface areas that allow larger fluxes of sulfate in to the cell through the cell membrane.

We recognize that cell sizes almost certainly cannot explain all of the variation between the subparallel ^34^ε–log_10_(csSRR) trends, and other factors such as the capacity for complete oxidation of organic substrates, growth temperature, and efficiency of sulfate transport may be important (Detmers et al., 2001; Canfield et al., 2006; Bradley et al., 2015). However, similarities amongst the normalized data for several *Desulfovibrio* species strongly indicate that biomass and cell sizes influence the observed ^34^ε in experimental and environmental studies. Ideally, we would compare our results to more of the previously published ^34^ε values reported for SRB and archaea. However, neither the original descriptions of the SRBs, nor previous isotopic studies of MSR provide detailed cell size measurements that are required for this type of analysis. Rather, they report cell size ranges and do not examine the dependence of the cell size on the growth condition. The broad variations in these ranges translate into a large range of cell volumes and do not allow meaningful comparisons with our data. We encourage future studies of MSR in pure cultures to report cell sizes or biomass as functions of growth condition.

### Fractionation of sulfur isotopes under phosphorus limitation

#### Morphological and physiological effects

Both *D. inopinatus* and DMSS-1 had slower growth rates (*k*) when limited by phosphate (Figure 4b and Table 3). The growth rate data for *D. inopinatus* are in good agreement with the Monod equation (Equation 7) (Monod, 1949) for a half-saturation constant (*K*_M_) of ~10 μM phosphate (Figure 4b). Growth rates for DMSS-1 are more scattered but also decrease below ~10 μM phosphate.

(7) $$k=k_{\text{max}}\frac{\left[{\text{PO}}_{4}^{3-}\right]}{K_{\text{M}}+[{\text{PO}}_{4}^{3-}]}$$

Phosphate limitation at ~10 μM for these species is similar to observations of phosphate limited growth at <12 μM for *D. desulfuricans* (Okabe and Characklis, 1992) and ~10 μM for *D. alaskensis* G20 (Bosak et al., 2016), suggesting that many *Desulfovibrio* species may use similar mechanisms to acquire phosphate during growth. Cellular yields (*Y*) decreased by several fold at lower phosphate concentrations (Table 3). Lower cell yields were also observed by Okabe and Characklis (1992) when the phosphate concentration was about 10 μM for *D. desulfuricans* grown in a chemostat; the C:P ratio of cells started to increase before the cell yield began to decrease. Thus, cells first adapted to moderately low phosphorus concentrations by reducing the cellular requirement for P. When the phosphorus availability decreased even further, cell yields began to decrease as well. Morphological changes observed in the two *Desulfovibrio* spp. we tested (see below), as well as structural adaptations to phosphate limitation and starvation observed in *D. desulfuricans* (Weimer et al., 1988) and *D. alaskensis* G20 (Bosak et al., 2016) are consistent with the higher bulk C:P ratio in phosphate-limited *D. desulfuricans* (Okabe and Characklis, 1992).

*D. inopinatus* and DMSS-1 cells elongated up to several hundred percent in the lowest phosphate conditions (Table 4). Cell elongation also occurs in phosphate limited cultures of *D. alaskensis* G20 (Bosak et al., 2016), albeit the overall effect in this microbe is smaller. Microscopic images of phosphate-limited *D. inopinatus* (Figure 2) revealed long chain-like cells as well as cells with more “typical” lengths. We attribute this cell morphology to incomplete cell division. Nucleic acids (particularly RNA, Elser et al., 2008; Yao et al., 2015) contain a very large fraction (up to tens of percent) of total cellular phosphorus; minimizing DNA and RNA synthesis and division may therefore enable survival in a competitive environment (see also Appendix). Growing phosphate-limited *D. alaskensis* G20 also replaces its membrane phospholipids by phosphorus-free lipids, synthesizes carbon-rich storage granules and metabolizes nucleic acids (Bosak et al., 2016). Cell elongation and the formation of intracellular carbon-rich granules may also aid in nutrient acquisition by increasing the surface area-to-volume ratio (Thingstad et al., 2005; Godwin and Cotner, 2015; Bosak et al., 2016). Also expected are decreases in the pools of phosphorus-containing metabolites dissolved in the cytoplasm: these metabolites should consist principally of inorganic phosphate, P_i_. Below, we discuss how a diminished cytoplasmic phosphorus content can influence the kinetics of enzymatic processes and influence the correlation between ^34^ε and csSRR.

#### Sulfur isotope fractionation

Phosphorus-limited *D. inopinatus* and DMSS-1 cultures grown on lactate did not fractionate sulfur isotopes very much (~5 to 8‰). These ^34^ε values were generally lower in phosphate-limited than in phosphorus-replete cultures (Figure 4a) and we did not observe a distinct correlation with csSRR. In contrast, ^34^ε values in DMSS-1 cultures grown in iron- and ammonium-limited media were ~5 and ~1‰ higher relative to the cultures grown in nutrient-replete media (Sim et al., 2012). The lower selectivity of the two phosphate-limited SRB species for sulfur isotopes may be related to any number of physiological changes experienced by cells at low phosphate concentrations: the composition and appearance of cell membranes and envelopes, the accumulation of storage polymers, transport of metals and others (Bosak et al., 2016). Some possibilities that directly influence the sulfate reducing pathway are discussed below.

**Figure 6:**
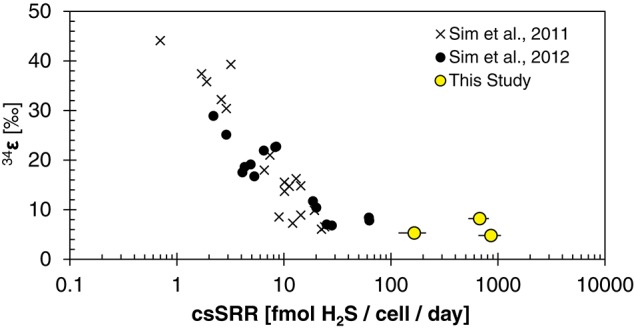
**Plot of ^34^ε and cell-specific sulfate reduction rate (csSRR) observed for DMSS-1 grown on various substrates (Sim et al., 2011b), and under nutrient-limited conditions [(Sim et al., 2012) and this study]**.

The fitted half-saturation constant (*K*_M_ = 10 μM, Figure 4) appears to coincide with the phosphate concentration at which the isotopic fractionation changes from <10‰ to >10‰ for *D. inopinatus*. Phosphate-limited DMSS-1 cultures exhibit ^34^ε–log_10_(csSRR) values that fall off and plot to the right (higher csSRR) of the trend observed by Sim et al. (2011b) (Figure 6). The large heterogeneity in cell lengths of *D. inopinatus* at low phosphate concentrations (Table 4 and Figure 2) complicates interpretations of csSRR, but assuming that per-cell enzyme activities scale linearly with either cell volume or surface area, the increases in cell size (up to threefold for DMSS-1, Table 4) related to phosphate depletion cannot explain up to tenfold higher csSRRs. The elevated rates may additionally reflect changes in biochemical kinetics within the cell.

**Figure 7:**
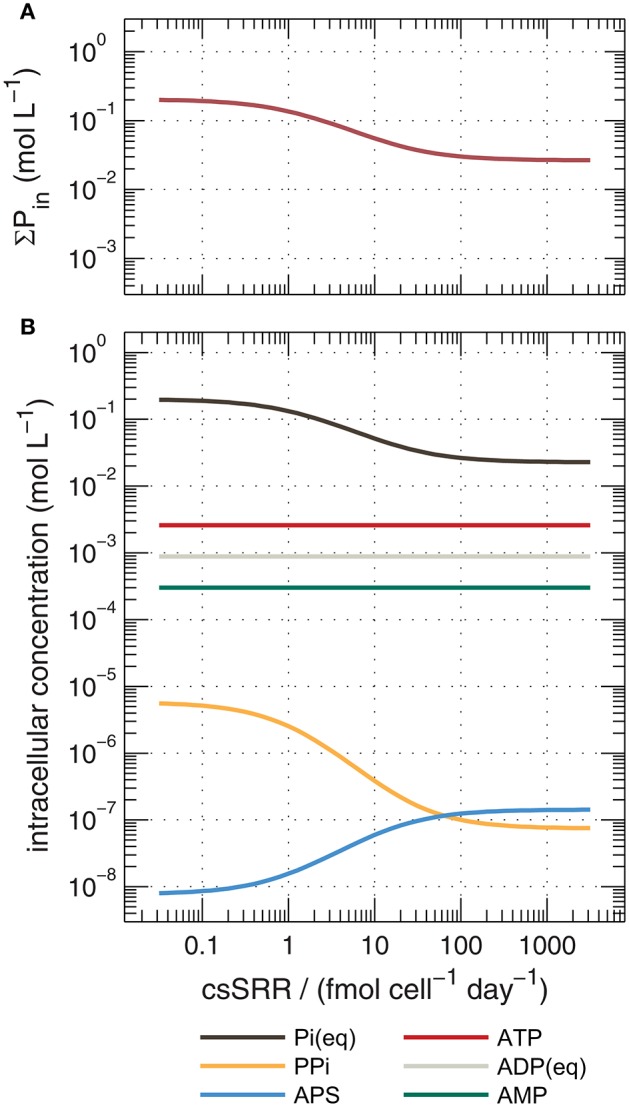
**Calculated concentrations of cytoplasmic phosphorus species during sulfate reduction by DMSS-1 predicted by an extended version of the model of Wing and Halevy (2014), described in the Appendix**. Shown are **(a)** total intracellular phosphorus (ΣP_in_, the sum of all species in panel, **b**), and **(b)** concentrations of individual species. Calculations assumed ${\left[{\text{SO}}_{4}^{2-}\right]}_{\text{out}}$ = 20 mM and [H_2_S] = 1 mM. P_i_, inorganic orthophosphate; PP_i_, pyrophosphate; APS, adenosine-5′-phosphosulfate; ATP, adenosine triphosphate; ADP, adenosine diphosphate; AMP, adenosine monophosphate.

Rees (1973) introduced the reversibility of steps in the respiratory chain as a control on overall sulfur isotope fractionation, and this concept has been invoked or extended to explain features of many experimental datasets (Trudinger and Chambers, 1973; Eckert et al., 2011; Sim et al., 2011a,b; Brunner et al., 2012; Antler et al., 2017). More recently, Wing and Halevy (2014) proposed a quantitative model of dissimilatory sulfate reduction that uses enzyme kinetic and thermodynamic data to calculate ^34^ε values as a function of csSRR. Their model explicitly calculates reversibilities, and thereby isotopic fractionations, for each enzymatic step in MSR. This approach offers insights into links between phosphorus, the sulfate reducing pathway and sulfur isotope fractionations. Phosphorus-containing metabolites (including the adenosine phosphates and inorganic pyrophosphate (PP_i_)) influence the thermodynamic drive of reactions in the respiratory chain of sulfate reducers (Equations A1–A4), and their cytoplasmic concentrations are either assumed or explicitly predicted in their model (Figure 7b). The major conclusion of Wing and Halevy's study is that concentrations of intracellular metabolites dictate the energetic favorability and reversibility (ratio of backwards to forwards fluxes) at each step, and influence the overall sulfate reduction rate and the expression of kinetic and equilibrium isotope effects.

**Figure A3:**
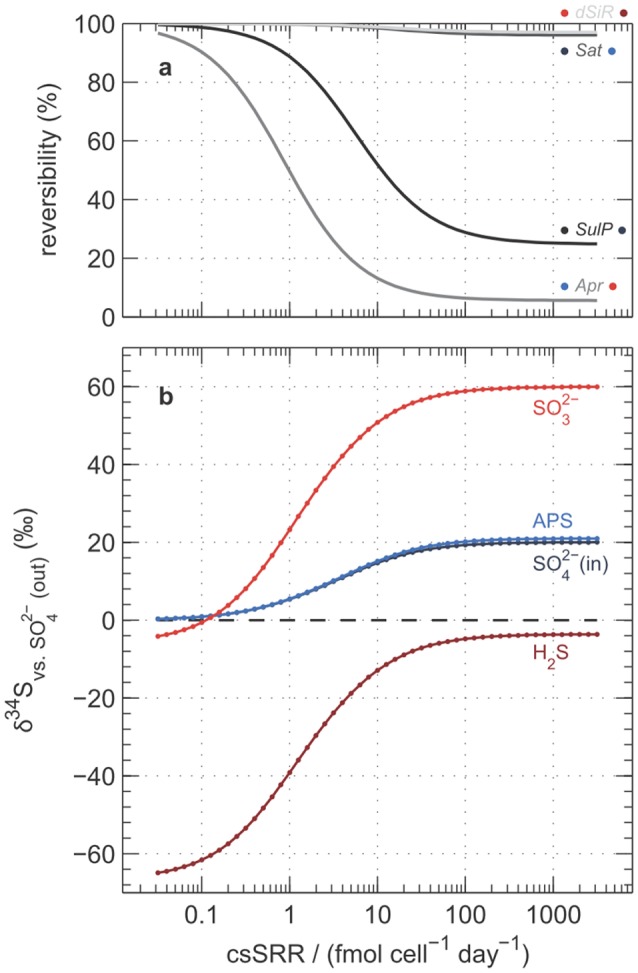
**Model predictions for (a)** reversibility of key reactions and **(b)** isotopic composition of intracellular sulfur compounds. Calculations follow Wing and Halevy (2014) and assumptions are identical to those used in Figure 7. Labels in panel **(a)** indicate the enzyme responsible (see Equations A1–A4), and coloring of dots to the left and right correspond to colors in **(b)** of the sulfur-containing reactants and products, respectively. Predicted δ^34^S values in **(b)** are expressed relative to extracellular sulfate at steady-state.

We reconstructed the numerical simulations of Wing and Halevy (2014), and extended them to simulate the changes in intracellular concentrations of orthophosphate (P_i_) and other phosphorus-containing metabolites as a function of csSRR. The most salient features of the model are described in the Appendix. Our model results suggest that high csSRRs are associated with lower phosphorus contents within the cytoplasm (Figure 7a). This is a direct consequence of Wing and Halevy's finding that reduction of adenosine-5′-phosphosulfate (APS) to sulfite (Equation A3) is the primary rate-limiting step in MSR under conditions probed by most experiments. Because [PP_i_] depends on [P_i_]^2^ (according to Equation A6), and because P_i_ accounts for most phosphorus that is dissolved in the cytoplasm (see discussion in Appendix), a small decrease in total cytoplasmic phosphorus during phosphorus-limited growth and starvation induces a large decrease in the PP_i_ concentration. Lowered PP_*i*_ levels are counterbalanced by an increase in the concentration of APS (because Equation A2, sulfate activation to APS, is always near equilibrium; *Sat* in Figure A3a), as shown in Figure 7b. The buildup of [APS] associated with lowered [P_i_] increases the thermodynamic drive for APS reduction (Equation A3). Intuitively, APS reduction becomes less reversible (*Apr* in Figure A3a), and therefore isotope fractionation between APS and the instantaneous sulfite product approaches the intrinsic kinetic isotope effect for this enzymatic step (22‰ in the default model of Wing and Halevy). Because the remaining step (sulfite reduction, Equation A4) is downstream of APS reduction and is almost fully reversible (*dSiR* in Figure A3a), the δ^34^S value of H_2_S is not sensitive to the kinetic isotope effect assumed for *dSiR* (in agreement with some experimental results, Leavitt et al., 2015, 2016) and is ~22‰ lower than that of APS when csSRR is high (Figure A3b).

**Figure A4:**
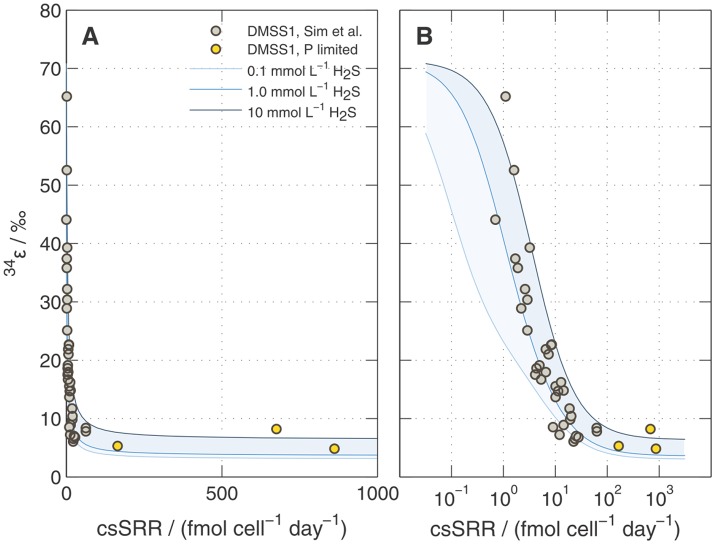
**Predicted ^34^ε during sulfate reduction by DMSS-1 following Wing and Halevy (2014)**. Calculations are shown for three different H_2_S concentrations (0.1, 1, and 10 mM) keeping [${\text{SO}}_{4}^{2-}$]_out_ constant at 20 mM. Overlaid on the model results are the data shown in Figure 6. Panels **(A)** and **(B)** show the same information but with linear and logarithmic x-axis scales (respectively).

Modeled ^34^ε values asymptote to a limit of ~5 ± 2‰ at high csSRRs, in good agreement with our data from DMSS-1 cultures (Figure A4). Several assumptions built into the model require further examination to determine if they are applicable under nutrient-limited conditions. In particular, this includes the parameterization of the scaling factor *u*_vivo−vitro_ (see Appendix). Although the agreement with our data may be circumstantial, the above treatment suggests a plausible mechanistic link between low extracellular phosphate concentrations and small ^34^ε both in the laboratory and in the environment. How phosphate limitation affects the fractionation of sulfur isotopes during growth on substrates that typically produce large fractionations (e.g., fructose) is an open question. The above analysis predicts a decrease in ^34^ε and increase in csSRR; a hypothesis that can be experimentally tested.

Studies of the sulfur cycle and the sulfur isotope record may benefit from consideration of the possible effects of phosphate limitation. While phosphorus is unlikely to limit growth of sulfate reducing bacteria in modern anoxic sediments where porewater phosphate concentrations are tens of micromolar or greater (Sundby et al., 1992), some studies hypothesize a more limited delivery flux of phosphate to Precambrian sediments (Bjerrum and Canfield, 2002; Reinhard et al., 2017). The very low sulfur isotope fractionation in sedimentary sulfates and sulfides during most of the Precambrian (Canfield, 1998) are consistent with this hypothesis. Overall, our data emphasize potential contributions of organic substrates and phosphate limitation to the persistently low sulfur isotope fractionations in the Archean and most of the Proterozoic.

## Conclusions

The magnitudes of sulfur isotope fractionation by the three studied species of *Desulfovibrio* scale with the negative logarithm of the cell-specific sulfate reduction rate, and the trend does not appear to depend on the particular organic substrate. These findings are in agreement with previous observations from pure cultures of several other species of sulfate reducing microorganisms and support the idea that the quality of organic substrates and the availability of nutrients, particularly those involved in energy conservation, are key factors in regulating the intracellular fluxes of sulfur compounds and the expression of sulfur isotope effects during dissimilatory sulfate reduction. Clear trends appear to link sulfur isotopic fractionation and microbial sulfate reduction rates, but with large spread in the data. Normalizing the sulfate reduction rates to the cell surface area or cell volume can reduce the spread and improve the correlation between sulfur isotope fractionation and sulfate reduction rate for several *Desulfovibrio* spp.

The cells of two species of SRB grown on lactate elongate and form chain-like cells during phosphate limitation (<10 μM initial phosphate), their cell-specific sulfate reduction rates increase relative to the phosphate-replete cultures, and their sulfur isotope fractionations approach ~5‰. Thus, sulfur isotope fractionation during microbial sulfate reduction in phosphorus-poor environments could deviate from predictions made under the assumption of unlimited phosphate supply. These experimental results are consistent with biochemical models that relate the kinetics and thermodynamics of enzyme-mediated reactions in the respiratory chain of SRB to expressed sulfur isotope effects.

## Author contributions

SO, TB, SZ, and DW: Designed the study. SZ: Carried out the majority of the analytical work, analyzed the data and wrote the first draft of the manuscript. DW: Carried out analytical work and modeling. All authors contributed ideas in the interpretation of the data and wrote the final manuscript.

### Conflict of interest statement

The authors declare that the research was conducted in the absence of any commercial or financial relationships that could be construed as a potential conflict of interest.

## Footnotes

^1^Cellular yield (*Y*) has units of cells per mole sulfate. It is a measure of the number of cells supported by a given amount of transferred electrons. Relating *Y* to biomass yield (i.e., g mol^−1^) requires accounting for cell size differences.

## Appendix

### Cellular distribution of phosphorus in SRB

Using literature data, we can roughly estimate the amounts of phosphorus contained in (i) metabolites dissolved in cytoplasm (ΣP_in_), (ii) in nucleic acids (DNA, RNA), and (iii) in lipid membranes of SRB cells. (i) Inorganic phosphate (P_i_), calculated from a biochemical model of dissimilatory sulfate reduction (described below), ranges between ca. 10^−2^ and 10^−1^ M intracellularly and comprises the majority of ΣP_in_ (Figure 7). This range is consistent with limited experimental data for other bacteria (Thauer et al., 1977, and references therein). A ~1 μm^3^ cell (= 1 × 10^−15^ L), typical of healthy DMSS-1 cells, would therefore contain between 0.01 and 0.1 fmol of ΣP_in_. Cells of other SRB we studied are typically 2–20 times larger (Table 4) and would contain proportionally more ΣP_in_. (ii) The phosphorus in genomic DNA can be estimated from the size of the bacterial genome. A cell with a ~3.6 Mbp genome, typical of *Desulfovibrio* spp. (Heidelberg et al., 2004; Hauser et al., 2011), would have 0.012 fmol P in its genomic DNA.^2^ Additional phosphorus is found within the nucleic acids in plasmids and RNA. RNA, in particular, can contain up to 25 times more phosphorus than genomic DNA in SRB (Postgate, 1979). (iii) Phospholipids can contain as much cellular phosphorus as genomic DNA (Van Mooy et al., 2009). The lipid phosphorus inventory is highly flexible, as evidenced by near-total replacement of phospholipids by phosphorus-free lipids in phosphate-limited SRB cultures (Bosak et al., 2016).

Total phosphorus content is the sum of these fractions (= ΣP_in_ + P_nucleic acids_ + P_lipids_) and can also be estimated independently from C:P ratios. For a ~1 μm^3^ cell, which is typical for healthy DMSS-1 cells (Table 4), and assuming a cellular carbon-to-volume ratio of 0.22 g C cm^−3^ (or 220 fg C μm^−3^; Bratbak and Dundas, 1984) and atomic C:P ratio of 100:1 (Fagerbakke et al., 1996)^3^ yields a total phosphorus content of 0.2 fmol P cell^−1^. This is similar to the sum of the above components. Due to the wide variation in cell size and C:P ratios amongst species and growth conditions, estimated inventories are probably accurate to no better than a factor of 10. A population of 1 × 10^7^ cells/ml would therefore require at least 2 μM phosphorus to synthesize all cellular components, a number that is of the same order of magnitude as phosphate concentrations (~10 μM) at which we and others have observed growth to be limiting in SRB cells.

### Intracellular phosphorus and sulfur isotope fractionation

We calculated intracellular concentrations of phosphorus and the sulfur isotope fractionation factor (^34^ε) as a function of csSRR, following the approach of Wing and Halevy (2014). Here, we briefly review key features of their numerical model, and then discuss some implications for phosphate metabolism in SRB.

Sulfate reduction is treated in four reversible enzymatically-mediated steps^4^ in the model of Wing and Halevy:

(A1) $${{\text{SO}}_{4}^{2-}}_{\text{out}}+n{{\text{H}}^{+}}_{\text{out}}⇌{{\text{SO}}_{4}^{2-}}_{\text{in}}+n{{\text{H}}^{+}}_{\text{in}}$$

(A2) $${{\text{SO}}_{4}^{2-}}_{\text{in}}+\text{ATP}⇌\text{APS}+{\text{PP}}_{\text{i}}$$

(A3) $$\text{ APS}+{\text{MK}}_{\text{red}}⇌{\text{SO}}_{3}^{2-}+{\text{MK}}_{\text{ox}}+\text{AMP}$$

(A4) $${\text{SO}}_{3}^{2-}{+\text{MK}}_{\text{red}}⇌{\text{H}}_{2}{\text{S}+\text{MK}}_{\text{ox}}$$

Enzymes that mediate the reactions in Equations A1–A4 are (respectively), sulfate permeases/transporters (SulP family), sulfate adenylyltransferase (Sat), APS reductase (Apr), and dissimilatory sulfite reductase (dSiR).

For given values of csSRR, the model yields predictions of steady-state intracellular concentrations of pyrophosphate (PP_i_), adenosine-5′-phosphosulfate (APS), and sulfite (${\text{SO}}_{3}^{2-}$). Several variables have to be specified. Those most relevant to our study are ${\left[{\text{SO}}_{4}^{2-}\right]}_{\text{out}}$, the extracellular sulfate concentration; [H_2_S], the sulfide concentration; concentrations of adenosine triphosphate (ATP) and adenosine monophosphate (AMP); and *u*_vivo–vitro_, a scaling factor to account for differences in enzyme concentrations between intact cells and cell extracts studied in enzyme kinetic experiments. The first two parameters can be constrained by batch culture experiments. ATP and AMP concentrations are held constant (at 2.6 and 0.3 mM, respectively), as are concentrations of the reduced/oxidized forms of the electron carrier (menaquinone, MK in Equations A3, A4). The remaining parameter *u*_vivo–vitro_ (intended as the ratio of enzyme activities in live SRB cells to enzyme activities in whole cell extracts) was calibrated by Wing and Halevy for DMSS-1 and several other species by fitting model results to isotopic data from pure cultures. We note that Wing and Halevy formulated *u*_vivo–vitro_ as a linear function of csSRR (i.e., *u*_vivo–vitro_ = *m* × csSRR + *b*, where *m* and *b* are constants chosen to fit ^34^ε–csSRR data for each species). Asymptotic behavior at high values of csSRR, where *b* ≪ *m* × csSRR, results from this parameterization. Although some evidence supports this scaling of gene expression with csSRR (Neretin et al., 2003; Wing and Halevy, 2014), it is unclear at present whether this relationship holds under phosphorus-limited conditions, as it would require high concentrations of enzymes under phosphorus stress. The biological reasons, if any, for this currently enforced asymptotic behavior requires further study.

Results of the model calculations are shown in Figures 7B, A3, A4. We noted several important features of the model results: (i) Most of the change in ^34^ε [the linear portion of the 1 mM H_2_S curve on the ^34^ε–log_10_(csSRR) plot in Figure A4B] occurs at csSRRs between 0.1 and 10 fmol cell^−1^ day^−1^; below this range, ^34^ε approaches its thermodynamic value of ~71‰; (Tudge and Thode, 1950), and above 10 fmol cell^−1^ day^−1^, ^34^ε asymptotically approaches a range between 3 and 7‰;. (ii) Over the range of csSRRs from 0.1 to 100 fmol cell^−1^ day^−1^], concentrations of PP_i_ decrease by 100-fold, and [APS] increases by 10-fold. (iii) Between 10 and 100 fmol cell^−1^ day^−1^, concentrations of PP_i_ and APS are still sensitive to changes in csSRR, but ^34^ε is not very sensitive (compare Figures 7B, A4B). This is because the reversibility of the process described by Equation A3 is insensitive to csSRR in this range (*Apr* in Figure A3a); the variation in ^34^ε with csSRR here is driven mostly by the changes in the reversibility of sulfate uptake (Equation A1), which retains some sensitivity to csSRR in this range (*SulP* in Figure A3a).

We extended the Wing and Halevy model to calculate inorganic phosphate (P_i_) and adenosine diphosphate (ADP) concentrations (Figure 7B). The model assumes that the concentrations of these species are governed by the following two equilibria (Thauer et al., 1977):

(A5) $$\text{ATP}+\text{AMP}⇌2\text{ADP},\;\Delta_{\text{r}}G{}^{\circ}\prime =0\text{ kJ }{\text{mol}}^{-1}$$

(A6) $${\text{PP}}_{i}+{\text{H}}_{2}\text{O}⇌2{\text{P}}_{i},\;\Delta_{\text{r}}G{}^{\circ}\prime =-21.9\text{ kJ }{\text{mol}}^{-1}$$

Because these reactions are catalyzed by efficient enzymes (adenylate kinase and pyrophosphatase, respectively; Cypionka, 1995), equilibrium is probably approached or attained under most growth conditions. Moderate deviations from the assumption of equilibrium do not influence the prediction of decreasing ΣP_in_ with higher csSRR shown in Figure 7A, because P_i_ makes up the majority of ΣP_in_ and exceeds the next most abundant species, ATP, by one or more orders of magnitude. In the model, decreased [P_i_] accommodates nearly the entirety of the decrease in ΣP_in_, leaving the concentrations of adenosine phosphates unchanged (Figure 7B); this behavior is consistent with observations that the adenylate energy charge of cells remains nearly constant over a range of physiological states (Thauer et al., 1977). However, absolute concentrations of P_i_, PP_i_, and APS are sensitive to order-of-magnitude variations in ${\left[{\text{SO}}_{4}^{2-}\right]}_{\text{out}}$ and [H_2_S] (Wing and Halevy, 2014). Of particular note is that [PP_i_] is highly sensitive to [P_i_] (Equation A6), so PP_i_ concentrations will be suppressed to very low levels when phosphate levels inside the cell are low. Because PP_i_ is a product of the reaction for sulfate activation by ATP (Equation A2), low [PP_i_] increases the thermodynamic favorability of this reaction and pulls it toward the right (lower reversibility).
